# Occlusal status of implant superstructures at mandibular first molar immediately after setting

**DOI:** 10.1186/s40729-015-0016-0

**Published:** 2015-07-04

**Authors:** Yukihiko Okada, Yuji Sato, Noboru Kitagawa, Keiichiro Uchida, Tokiko Osawa, Yoshiki Imamura, Mayumi Terazawa

**Affiliations:** Department of Geriatric Dentistry, School of Dentistry Showa University, 2-1-1 Kitasenzoku, Ota Ward, Tokyo 145-8515 Japan

**Keywords:** Occlusal load, Occlusal contact area, Implant, Clenching strength, Occluzer, BiteEye

## Abstract

**Background:**

Occlusal contact on the implant superstructures is important for successful treatment. The purpose of this study was to investigate the occlusal contact of single implant superstructures at the mandibular first molar immediately after seating from weak to strong clenching.

**Methods:**

Subjects were nine patients who had just been fitted with an implant prosthesis in the mandibular first molar region, with no missing teeth other than in the implant region. First, while masseter muscle activity was monitored, maximum clenching strength (100 % maximum voluntary contraction (MVC)) was determined with an electromyogram. Next, occlusal load and occlusal contact area were measured three times at clenching intensities of 40, 60, 80, and 100 % MVC by the use of pressure-sensitive film for occlusal force diagnostic and Occluzer for occlusal force measurement. Finally, the occlusal contact area was measured once each at 20, 40, and 60 % MVC using a silicone testing material and BiteEye for occlusal contact measurement. A two-way analysis of variance (ANOVA) was used to determine occlusal loading and occlusal area as dependent variables, and clenching strength and presence or absence of implant as between-subject factors. A multiple comparison test was performed using the Bonferroni method.

**Results:**

The occlusal contact area and occlusal load of the implant prosthesis increased with clenching strength, and the increases in occlusal contact area and occlusal load of the implant prosthesis were less than those of the contralateral tooth at high clenching strength. However, significant difference was not observed when compared with both sides of the molar region regardless of clenching strength.

**Conclusions:**

The occlusal contact area of the implant had a tendency to be adjusted smaller than the natural tooth by a dental technician and a dentist. On the other hand, despite the small tissue displaceability of the implant, occlusal load on the implant prosthesis was smaller than on the natural tooth at high clenching strength.

## Background

Implants maintain osseointegration with the inside of the bone over long periods of time, and mechanical factors are important for retaining function [1]. Particularly, when natural teeth and implants are present together, differences in tissue displaceability when they are subjected to occlusal force may cause dynamic imbalance from strong to weak clenching [2–4].

Therefore, there is a school of thought that prosthetic implants should be given a lower occlusion than natural teeth [5–7]. On the other hand, from the perspective of jaw function, it is also argued that prosthetic implants should be given the same occlusion as intact natural teeth neighboring the implant [8]. In a study using two-dimensional finite element analysis, Maezawa et al. [9] suggested that, even if the occlusal surface of the prosthetic implant is made lower than the occlusal plane, high clenching intensity can result in an excessive occlusal load on the prosthetic implant. In addition, Koyama et al. [10] found no significant differences in occlusal contact point between prosthetic implants and natural teeth, even if clenching intensity varies. From these studies, it appears that the occlusal contact of implant prostheses is the same level on natural teeth, regardless of clenching strength, and that occlusal load on implant prosthesis increases more than contralateral natural teeth as clenching strength increases. Methods of quantitative evaluation used to clarify mechanical factors of implant prostheses include pressure-sensitive films for occlusal force diagnostic use and silicone testing materials, but few studies have examined the occlusal contact from weak to strong clenching strength under the same conditions.

In this department to date, Okuyama et al. [11] used a pressure-sensitive film to examine the occlusal contact and to calculate the mean occlusal gap and occlusal load of implants that had progressed satisfactorily, as well as natural teeth. Imamura et al. [12] used a new silicone test material and a pressure-sensitive film to develop a method for investigating changes in occlusal contact from weak to strong clenching intensity in subjects with natural dentition.

Occlusal contact is a reflection of the degree of functional recovery and measurement of factors such as number of occlusal contact points, contact area, and distribution. Occlusal center is advisable before and after treatment [13]. Therefore, the purpose of this study was to investigate the occlusal contact of single implant superstructures at the mandibular first molar immediately after seating by using two different materials with an electromyogram. Ultimately, we aimed to obtain clinical suggestions for the occlusion that should be given to prosthetic implants through a longitudinal follow-up study.

## Methods

### Subjects and dentition

Subjects were nine outpatients (5 male, 4 female) attending Showa University Dental Hospital, aged 35–69 years (mean age, 49 ± 12 years) (Table 1). All subjects were cases of single implants placed at the mandibular first molar deficit, on the day of implant prosthesis setting and occlusal adjustment. The criteria for subject selection were no inflammatory symptoms (redness, swelling) in the implantation area [14, 15], no implant mobility, no subjective symptoms, and no noticeable resorption on X-ray photographs.

**Table 1:** Site of implants

No.	Sex	Age	Implant system	Implant site
1	F	35	Straumann	Right
2	F	39	Brånemark	Left
3	F	47	Brånemark	Left
4	F	49	Brånemark	Left
5	M	43	Straumann	Right
6	M	46	Straumann	Right
7	M	69	Straumann	Right
8	M	48	Brånemark	Left
9	M	67	Brånemark	Right

The requirements for dentition were that other than the implant, which was the tooth to be studied, all teeth were natural; 28 teeth were present from the central incisors to the second molars, which includes the implant area; there were no mobile teeth; a basic periodontal test showed no pockets of 4 mm or more; there was no history of orthodontic therapy; and there was no oromandibular dysfunction such as temporomandibular disorder, masticatory muscle pain, or mandibular movement abnormality. In addition, occlusal adjustment was performed by a doctor in attendance. The implant prosthesis had one or more occlusal contact points at maximum clenching strength. Moreover, it has been adjusted as not to interfere with existing guide during lateral movement. Fourteen patients were selected based on these conditions, and consent was obtained from the doctor in charge of the 12 patients. Of these, nine patients consented to take part and acted as subjects.

The study was approved by Showa University Ethics Committee and was carried out after all subjects received a full explanation of the aims and methods of the study and gave their consent to participate (approval no. 2012-020).

### Electromyograph attachment

Masseter muscle activity was measured using an electromyograph (PowerLab; ADInstruments, Nagoya, Japan). Silver disk electrodes of 10 mm in diameter with bipolar leads (Duotrode; Morita Corp., Osaka, Japan) were adhered on both sides of the central part of the masseter muscle. The distance between electrodes was 21 mm, and the electrodes were placed parallel to the direction in which the masseter muscle fibers run.

The activity of the masseter muscle, when subjects clenched their teeth at full strength with nothing interposed between the upper and lower teeth, was defined at 100 % maximum voluntary contraction (MVC), and subjects were able to see the amount of muscle force displayed in numerical values through visual feedback.

### Measurement and analysis of subject dentition

#### Measurement of occlusal loading and occlusal contact area using Occluzer

Pressure-sensitive film (Dental Prescale 50H type R; Fuji Photo Film Co., Tokyo, Japan) for occlusal force diagnostic use was used to examine the occlusal contact area and occlusal load in the intercuspal position together. Masseter muscle activity (clenching strength) was set at 40, 60, 80, and 100 % MVC, and subjects were measured three times at each of these clenching intensities using visual feedback. Subjects remained in a seated position, and the head on the headrest of the dental unit with the occlusal plane is parallel to the floor.

Subjects were instructed to open their mouths one finger width and the Prescale was inserted, and subjects were then instructed to slowly close their mouths and clench their teeth. The Prescale was interposed between the full dentition between the second molars on either side, and subjects were instructed to bite in the intercuspal position. Clenching on the Prescale was carried out for 3 s, and considering the content of muscle fatigue, measurements were taken at 5-min intervals.

The Prescale was kept in a cool, dark place for 24 h; after which, the colored parts, their surface area, and their color density were analyzed using a dedicated analyzer (Occluzer FPD707®; Fuji Photo Film Co., Tokyo, Japan). Occlusal force was analyzed by a software (DePROS-PC; GC, Tokyo, Japan), and these data were converted to pressure values. The occlusal loading and occlusal contact area for molars were then calculated.

#### Measurement of occlusal contact area using BiteEye

A material for checking accuracy of fit (Blue Silicone®; GC, Tokyo, Japan) was used to examine the occlusal contact in the intercuspal position. The occlusal contact area was measured once at each clenching strength (20, 40, and 60 % MVC) using visual feedback. Subject posture was the same as during Prescale measurement. Subjects were instructed to open their mouths one finger width and Blue Silicon was inserted above the lower row of teeth, and subjects were then instructed to keep it in the mouth for 15 s. Subjects were instructed to keep the clenching strength of provisions while displaying the strength clenching on the monitor. Subjects clenched their teeth at the required strength for 30 s and kept the Blue Silicone in their mouths for a further 30 s until it hardened. Taking muscle fatigue into account, measurements were taken at 5-min intervals.

Thus, the Blue Silicone impressions obtained were examined using a BiteEye occlusal contact analyzer (BiteEye-I®; GC, Tokyo, Japan). The thickness of the Blue Silicone film at the points of occlusal contact was set at 10, 20, and 30 μm, and the occlusal contact area was calculated. When the thickness of the Blue Silicone film at the points of occlusal contact was set at 10 μm, Dental Prescale and Blue Silicone approximate in the occlusal contact area in the natural dentition [12].

While there have been studies in which the occlusal contact area was calculated from the silicone method, the thickness of silicone (μm) that is defined as occlusion has not been quantitatively clarified. Okada et al. [16] found functional occlusal contacts can be recorded at the silicone thickness 30 μm. From the above sentence, the thickness of the Blue Silicone film at the points of occlusal contact was set at 10–30 μm.

#### Analysis of the results from Occluzer and BiteEye

The following calculations were performed:

1. Comparison of Occluzer and BiteEye in the occlusal contact area
2. Comparison of the occlusal contact area and occlusal load between the implant and same number of natural teeth on the contralateral side
3. Comparison of the occlusal contact area and occlusal load between molar areas of the implant side and contralateral side
4. Comparison of the occlusal contact area and occlusal load between molar areas of the implant side and contralateral side excluding the first molar
5. Comparison of the proportion of the occlusal contact area and occlusal load on the molar region accounted for by the prosthetic implant and contralateral tooth

Analysis of results from Occluzer and BiteEye was performed by two-way ANOVA with occlusal loading and occlusal area as dependent variables, and clenching strength and presence or absence of implant as between-subject factors. The level of significance for multiple comparison tests was set at 5 %.

In addition, multiple comparison test was performed using the Bonferroni method. PASW Statistics 18 was used for all statistical calculations (SPSS, Tokyo, Japan).

## Results

### Comparison of occlusal contact areas evaluated using Occluzer and BiteEye

**Fig. 1:**
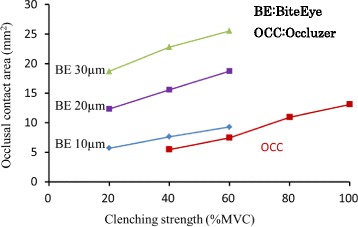
Comparison of the occlusal contact area between Occluzer and BiteEye

Occlusal contact area values obtained with BiteEye and Occluzer at clenching intensities from 20 to 100 % MVC were compared (Fig. 1). At different clenching intensities, the contact area values of BiteEye with silicone thickness of 10 μm (BE 10 μm) were the most similar to the contact area values of Occluzer. Thus, comparisons of occlusal contact area values between Occluzer and BiteEye were carried out with a silicone thickness of 10 μm.

### Comparison of implant region and contralateral tooth

#### Comparison of occlusal contact area

**Fig. 2:**
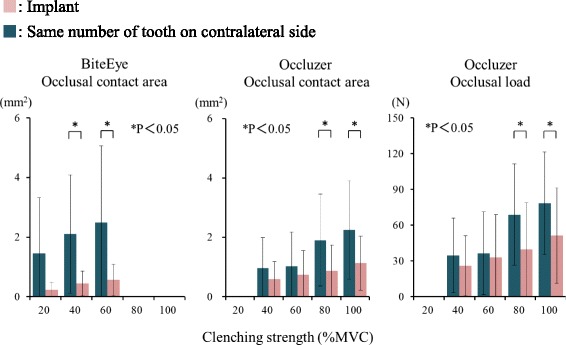
Comparison of occlusal contact area and occlusal load between implant and contralateral tooth

The occlusal contact area of implant prostheses increased with clenching strength and was always less than that of the contralateral tooth (Fig. 2). ANOVA results (Table 2) show a significant difference between implants and natural teeth with BiteEye (*P* < 0.05). Multiple comparison test showed that the occlusal contact area of implants was significantly smaller than the occlusal contact area of contralateral teeth with BiteEye at clenching intensities of 40 and 60 % MVC and Occluzer at clenching intensities of 80 and 100 % MVC (*P* < 0.05).

**Table 2:** Two-way ANOVA of the occlusal contact area of the implant prosthesis and contralateral tooth

	Source	Sum of square	*df*	Mean square	*F* value	*P* value
Occluzer	Implant or natural tooth	1941.060	1	1941.060	1.074	0.333
	MVC	424.098	3	141.336	1.958	0.147
	Interaction	262.581	3	87.527	1.345	0.283
BiteEye	Implant or natural tooth	28.022	1	28.022	6.892	0.030
	MVC	3.374	2	1.687	1.927	0.178
	Interaction	0.916	2	0.458	0.827	0.455

#### Comparison of occlusal load

Occlusal load on implant prostheses increased with clenching strength and was always less than that on the contralateral teeth (Fig. 2). At 100 % MVC, the occlusal load on the implant prosthesis was 47.7 ± 39.0 N and that on the contralateral tooth was 81.4 ± 41.1 N. ANOVA results (Table 3) showed a significant interaction in the difference in occlusal load between implants and natural teeth, and clenching strength (*P* < 0.05). Multiple comparison test showed that the occlusal load was significantly smaller on the implant prostheses than on the contralateral teeth at clenching intensities of 80 and 100 % MVC (*P* < 0.05).

**Table 3:** Two-way ANOVA of occlusal load of implant prosthesis and contralateral tooth

Source	Sum of square	*df*	Mean square	*F* value	*P* value
Implant or natural tooth	8732.252	1	8732.252	13.299	0.007
MVC	14,619.565	3	4872.855	16.724	0.001
Interaction	2200.789	3	733.596	8.442	0.001

The rate of increase in occlusal load from 40 to 100 % MVC was 106.1 % for the implant prosthesis and 127.4 % for the contralateral tooth.

### Comparison of implant side and contralateral side molar regions

#### Comparison of occlusal contact area in the molar region

**Fig. 3:**
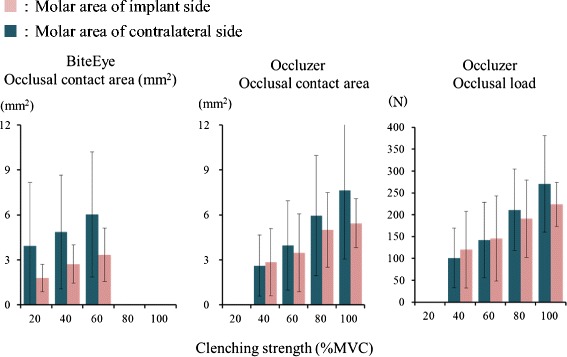
Comparison of the occlusal contact area and occlusal load between the implant side molar region and contralateral side molar region

The occlusal contact area in the molar region on the implant and contralateral sides increased with clenching strength and, at clenching intensity of 60 % MVC or above, was less on the implant side molar region (Fig. 3). ANOVA results (Table 4) showed no significant differences in occlusal contact area between the implant side molar region and the contralateral side molar region with BiteEye (*P* = 0.092). Multiple comparison tests showed no significant differences in the occlusal contact area between the molar region on the implant side and the molar region on the contralateral side at any clenching intensity with either BiteEye or Occluzer (*P* > 0.05).

**Table 4:** Two-way ANOVA of the occlusal contact area of the implant side molar region and contralateral side molar region

	Source	Sum of square	*df*	Mean square	*F* value	*P* value
Occluzer	Implant side and contralateral side	24.453	1	24.453	2.334	0.165
	MVC	159.749	3	53.253	26.918	0.000
	Interaction	16.135	3	5.378	3.786	0.024
BiteEye	Implant side and contralateral side	51.431	1	51.431	3.674	0.092
	MVC	32.471	2	16.236	8.125	0.004
	Interaction	0.236	2	0.118	0.072	0.931

#### Comparison of occlusal load on the molar region

Occlusal load on the implant side molar region and the contralateral side molar region increased with clenching strength and, at clenching intensity of 80 % MVC and above, was less on the implant side molar region (Fig. 3). At 100 % MVC, the occlusal force on the implant side molar region was 212.7 ± 57.6 N and on the contralateral side molar region was 274.4 ± 111.5 N. ANOVA results (Table 5) showed no significant differences in occlusal load between the implant side molar region and the contralateral side molar region (*P* = 0.278). Multiple comparison test showed no significant differences in occlusal load between the molar region on the implant side and the molar region on the contralateral side at any clenching intensity (*P* > 0.05).

**Table 5:** Two-way ANOVA of occlusal load of the implant side molar region and contralateral side molar region

Source	Sum of square	*df*	Mean square	*F* value	*P* value
Implant side and contralateral side	8578.099	1	8578.099	1.354	0.278
MVC	193,888.518	3	64,629.506	36.593	0.000
Interaction	30,575.358	3	4016.245	3.153	0.430

### Comparison of implant side and contralateral side molar regions excluding the first molar

#### Comparison of occlusal contact area in the molar region excluding the first molar

**Fig. 4:**
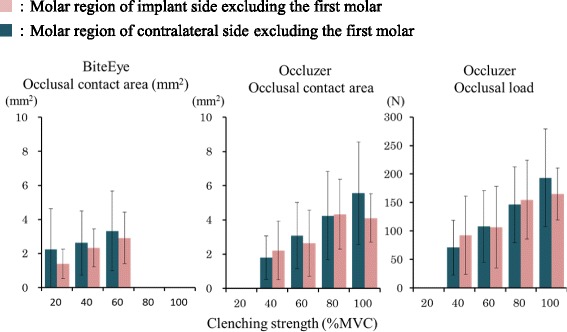
Comparison of the first molar-eliminated occlusal contact area and load between the implant side molar region and contralateral side molar region

The occlusal contact area in the molar region excluding the first molar on the implant and contralateral sides increased with clenching strength (Fig. 4). ANOVA results (Table 6) showed no significant differences in the occlusal contact area between the implant side molar region and the contralateral side molar region with BiteEye (*P* = 0.400). Multiple comparison tests showed no significant differences in the occlusal contact area between the molar region excluding the first molar on the implant side and the molar region on the contralateral side at any clenching intensity with either BiteEye or Occluzer (*P* > 0.05).

**Table 6:** Two-way ANOVA of the occlusal contact area of the implant side molar region and contralateral side molar region excluding the first molar

	Source	Sum of square	*df*	Mean square	*F* value	*P* value
Occluzer	Implant side and contralateral side	2.177	1	2.177	0.383	0.165
	MVC	90.753	3	30.251	25.800	0.553
	Interaction	9.011	3	3.004	2.209	0.113
BiteEye	Implant side and contralateral side	3.527	1	3.527	0.789	0.400
	MVC	15.013	2	7.541	10.636	0.001
	Interaction	0.790	2	0.395	0.661	0.530

#### Comparison of occlusal load on the molar region excluding the first molar

Occlusal load on the implant side molar region and the contralateral side molar region excluding the first molar increased with clenching strength (Fig. 4). At 100 % MVC, the occlusal force on the implant side molar region was 165.0 ± 45.8 N and on the contralateral side molar region was 193.3 ± 85.8 N. ANOVA results (Table 7) showed no significant differences in occlusal load between the implant side molar region and the contralateral side molar region (*P* = 0.990). Multiple comparison test showed no significant differences in occlusal load between the molar region excluding the first molar on the implant side and the molar region on the contralateral side at any clenching intensity (*P* > 0.05).

**Table 7:** Two-way ANOVA of occlusal load of the implant side molar region and contralateral side molar region excluding the first molar

Source	Sum of square	*df*	Mean square	*F* value	*P* value
Implant side and contralateral side	0.690	1	0.690	0.000	0.990
MVC	102,482.810	3	34,160.937	30.344	0.000
Interaction	6049.786	3	2016.595	1.336	0.286

### Comparison of proportion of occlusal contact area and occlusal load on the molar region accounted for by prosthetic implant and contralateral tooth

**Fig. 5:**
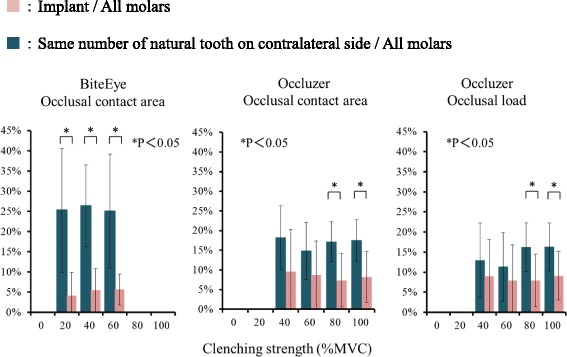
Proportion of the occlusal contact area and occlusal load of the whole molar region accounted for by the implant prosthesis and by the contralateral tooth

With both BiteEye and Occluzer, the proportion of the occlusal contact area on the molar region overall accounted for by the prosthetic implant was less than the proportion accounted for by the contralateral natural tooth, and ANOVA showed this difference to be significant (*P* < 0.05) (Fig. 5, Table 8). Multiple comparison test also showed that the prosthetic implant accounted for a significantly smaller proportion of occlusal contact area at all clenching intensities other than 40 and 60 % MVC with Occluzer (*P* < 0.05). With occlusal load as well, the prosthetic implant accounted for a significantly smaller proportion than the contralateral tooth, and at 100 % MVC, the occlusal load on the prosthetic implant was 9.1 % of the load on the molar region while the occlusal load on the contralateral tooth was 16.3 %. Multiple comparison test showed that the proportion of occlusal load on the molar region accounted for by the prosthetic implant was significantly smaller than that accounted for by the contralateral tooth at clenching intensities other than 40 and 60 % (*P* < 0.05).

**Table 8:** Two-way ANOVA of the proportion of occlusal load and contact area for implants and first molars based on molar area

	Source	Sum of square	*df*	Mean square	*F* value	*P* value
Occlusal load						
Occluzer	Implant or natural tooth	585.390	1	585.390	9.870	0.014
	MVC	53.901	3	53.901	1.768	0.220
	Interaction	46.872	3	46.872	3.791	0.870
Occlusal contact area						
Occluzer	Implant or natural tooth	0.087	1	0.087	11.101	0.010
	MVC	0.003	3	0.003	1.202	0.305
	Interaction	0.004	3	0.004	2.178	0.178
BiteEye	Implant or natural tooth	0.291	1	0.291	12.210	0.008
	MVC	0.040	2	0.040	1.287	0.290
	Interaction	0.000	2	0.000	0.070	0.798

No significant differences in the proportion of occlusal area or occlusal load on the molar region attributable to clenching strength were found with the prosthetic implant or the contralateral tooth with either BiteEye or Occluzer (*P* > 0.05) (Table 8).

## Discussion

### Subjects and dentition

Males outnumbered females in the present study, but there have been no reports of differences in mean occlusal load between males and females in the 50–54 age group [17]. While there have been reports that in healthy, dentulous subjects, the total occlusal force in the molar region on one side is approximately 400 N at maximum clenching strength [18], in the present study, the mean occlusal force on the natural tooth side at 100 % MVC was 274.4 ± 111.5 N. However, considering the subjects in the present study were of middle and old age, with mean age 49 years, the results probably have a certain validity [17–19].

Occlusal force is often measured across the whole jaw [20, 21]. The Dental Prescale used for occlusal force measurement has a thickness of approximately 100 μm, and concern has been raised that this thickness might affect the results of occlusal force distribution measurement. However, as good reproducibility of results from the molar region has been reported, in the present study, the molar region from the first premolar to the second molar on both sides was examined [11, 12, 22].

### Study methods

#### Examination of occlusal contact

Materials such as wax, occlusal registration paper, and pressure-sensitive paper are used to examine occlusal contact. The present study utilized an examination method using silicone [11, 12, 16]. The Blue Silicone used in the present study is a cartridge type, which allows a nearly constant mixing ratio to be maintained, it has low viscosity and thus flows well, and it shows little dimensional change over time. It therefore fully met the requirements for a material for registration of occlusal contact status.

A prior study reported low reproducibility when using Dental Prescale at low clenching strength [22]. In addition, the use of Blue Silicone to take impressions at high clenching intensities of 80 and 100 % MVC places a burden on subjects, as Blue Silicone takes a long time to harden. For these reasons, when measurements from low to high clenching strength were made in the present study, Blue Silicone was used at clenching intensities of 20, 40, and 60 % MVC, and Dental Prescale was used at 40, 60, 80, and 100 % MVC.

#### Measurement of occlusal load

Occlusal load was measured with Dental Prescale and analyzed with Occluzer. The Dental Prescale system allows occlusal contact pressure to be measured quickly and easily across the dentition, and it is of enormous clinical utility because its accuracy and reproducibility in the molar region have been confirmed [16, 20, 22]. The technique using this sequence and also the method of cross-checking occlusal contact points using silicone and Dental Prescale are clinically useful and have been widely studied and reported [16, 20].

### Comparison of occlusal contact areas evaluated using Occluzer and BiteEye

Blue Silicone with a thickness of 10 μm and Dental Prescale gave similar values for the occlusal contact area at 40 and 60 % MVC. These results probably have more validity than those reported by Imamura et al. [12].

### Analysis of implant region

#### Occlusal load on implant region

An occlusal load of approximately 130 N on the first molar has been reported at maximum clenching strength in healthy, dentulous subjects [18]. In the present study, occlusal load on the mandibular first molar was somewhat less, at 81.2 ± 41.1 N. However, bearing in mind that the subjects in the prior study were in their 20 s whereas those in the present study were of middle to old age, with a mean age of 49 years, the present results are probably somewhat valid. Implant prostheses probably have greater occlusal load than natural teeth at higher clenching intensities because they lack the mechanical buffering function of the periodontal membrane. A study using two-dimensional finite element analysis on the mandibular first molar by Maezawa et al. [9] suggests that even if the occlusal surface of the prosthesis is made lower than the occlusal plane, the implant area may still be subjected to excess occlusal load with increased clenching strength.

In the present study, however, the occlusal load on the implant prosthesis tended not to increase as much as the load on the contralateral tooth when the clenching strength was higher. A possible reason is that the dentists adjusted the implant prostheses with pressure displacement in mind, giving a smaller occlusal contact area so that there were fewer loading points than in the contralateral tooth [4, 21, 22].

When considering the balance of occlusal load in the molar region, it is better to give the same occlusal load on both molar regions at 100 % MVC [12, 16]. However, in this study, the occlusal load was significantly smaller on the implant prosthesis than on the contralateral tooth at 100 % MVC.

#### Measurement of occlusal contact area of implant prosthesis

Dental Prescale has a thickness of approximately 100 μm, whereas Blue Silicone has less thickness and is therefore likely to give more accurate measurements of the occlusal contact area [12, 23]. In addition, when the occlusal contact areas of implant prostheses and their contralateral teeth were compared, the occlusal contact area of the contralateral teeth was significantly greater at higher clenching strength. This is probably because occlusion between natural teeth results in greater displacement due to the presence of a periodontal membrane on both teeth. In addition, Koyama et al. [10] reported no significant differences between the molar region on the implant side and on the contralateral side, even when clenching strength varied. Similarly, when clenching strength varied in this study, the occlusal contact area of the implant prosthesis did not increase than the contralateral tooth.

#### Comparison of proportion of occlusal contact area and occlusal load in all teeth accounted for by prosthetic implant and contralateral tooth

The proportion of occlusal force on the molar region of healthy dentition accounted for by the mandibular first molar on one side has previously been reported as 16 % [20]. In the present study, however, the implant prosthesis accounted for a lower proportion of 9 % of the occlusal force on the molar region. No significant differences in occlusal load were observed between sides, but the proportion of occlusal load on all teeth borne by the implant prosthesis was less than that borne by the contralateral tooth. However, there was no significant difference in occlusal load between both molar regions. It suggested that occlusal loading on both molar regions have been balanced.

### Future research

The present study examined implant prostheses immediately after setting, but the occlusal contact of implant prostheses is believed to change over time as a result of factors such as extrusion of opposing teeth, abrasion from neighboring surfaces, and tooth attrition. In the future, we intend to use the results of the present study to carry out a prospective study that will survey the period of 1 year from immediately after setting, which is when problems are most common. This future study will have a greater number of subjects and will evaluate parameters such as mastication function. This study will aim to clarify how the occlusal contact of the implant prosthesis changes within the dentition and to draw up guidelines on this basis.

## Conclusions

The results suggest that the occlusal contact of implant prostheses can be evaluated from low to high clenching intensities using Blue Silicone and Dental Prescale. There was a trend for implant prostheses to be adjusted such that immediately after setting the occlusal contact area, the occlusal load of the implant superstructure was less than that of the contralateral tooth. This is likely to be due to dentists taking into account the small tissue displaceability of implants.

The occlusal load on implant prostheses in a single intermediary mandibular first molar deficit tended to increase less with clenching strength than the load on the contralateral tooth. In addition, the proportion of the occlusal load on the whole dentition accounted for by the implant prosthesis was less than the proportion accounted for by the contralateral tooth. However, there was no significant difference in occlusal load between both molar regions. It suggested that occlusal loading on both molar regions has been balanced. The adequate occlusion on implant prosthesis has not been clear. However, we will be able to obtain clinical suggestions for the occlusion that should be given to prosthetic implants through a longitudinal follow-up study.

## Abbreviations

MVC: maximum voluntary contraction
Prescale: Dental Prescale 50H type R
Occluzer: Occluzer FPD707
BiteEye: BiteEye-I
ANOVA: analysis of variance
